# Projected impact of population aging on non-communicable disease burden and costs in the Kingdom of Saudi Arabia, 2020–2030

**DOI:** 10.1186/s12913-023-10309-w

**Published:** 2023-12-08

**Authors:** David C. Boettiger, Tracy Kuo Lin, Maram Almansour, Mariam M Hamza, Reem Alsukait, Christopher H. Herbst, Nada Altheyab, Ayman Afghani, Faisal Kattan

**Affiliations:** 1 Institution for Health and Aging, University of California, San Francisco, CA 94158 USA; 2 The Ministry of Economy and Planning, Riyadh, Saudi Arabia; 3 Nutrition and Population Global Practice, World Bank, Washington, D.C USA; 4 Community Health Sciences, King Saud University, Riyadh, Saudi Arabia

**Keywords:** Aging, Non-communicable Disease, Costs, Saudi Arabia

## Abstract

**Background:**

The number of people aged greater than 65 years per 100 people aged 20–64 years is expected to almost double in The Kingdom of Saudi Arabia (KSA) between 2020 and 2030. We therefore aimed to quantify the growing non-communicable disease (NCD) burden in KSA between 2020 and 2030, and the impact this will have on the national health budget.

**Methods:**

Ten priority NCDs were selected: ischemic heart disease, stroke, type 2 diabetes, chronic obstructive pulmonary disease, chronic kidney disease, dementia, depression, osteoarthritis, colorectal cancer, and breast cancer. Age- and sex-specific prevalence was projected for each priority NCD between 2020 and 2030. Treatment coverage rates were applied to the projected prevalence estimates to calculate the number of patients incurring treatment costs for each condition. For each priority NCD, the average cost-of-illness was estimated based on published literature. The impact of changes to our base-case model in terms of assumed disease prevalence, treatment coverage, and costs of care, coming into effect from 2023 onwards, were explored.

**Results:**

The prevalence estimates for colorectal cancer and stroke were estimated to almost double between 2020 and 2030 (97% and 88% increase, respectively). The only priority NCD prevalence projected to increase by less than 60% between 2020 and 2030 was for depression (22% increase). It is estimated that the total cost of managing priority NCDs in KSA will increase from USD 19.8 billion in 2020 to USD 32.4 billion in 2030 (an increase of USD 12.6 billion or 63%). The largest USD value increases were projected for osteoarthritis (USD 4.3 billion), diabetes (USD 2.4 billion), and dementia (USD 1.9 billion). In scenario analyses, our 2030 projection for the total cost of managing priority NCDs varied between USD 29.2 billion - USD 35.7 billion.

**Conclusions:**

Managing the growing NCD burden in KSA’s aging population will require substantial healthcare spending increases over the coming years.

**Supplementary Information:**

The online version contains supplementary material available at 10.1186/s12913-023-10309-w.

## Background

Life expectancy at birth in The Kingdom of Saudi Arabia (KSA) increased from 70.5 years in 2000 to 74.3 years in 2019 [1]. The number of people aged greater than 65 years per 100 people aged 20–64 years is expected to almost double in KSA between 2020 and 2030 (from 5.3 to 9.5 per 100) [2]. The impact of the world’s aging population on healthcare systems is a matter of international concern and a top priority for KSA. KSA’s economy is also dependent on a few key sectors (e.g., crude oil production, petroleum refining) and a large migrant labor workforce, making it vulnerable to shifts within domestic and global economies. It is critical for KSA to consider how population aging will impact the economic demands of its healthcare system in the future.

A key issue is the management of KSA’s growing burden of age-associated non-communicable disease (NCD). In 2019, NCDs accounted for 67.4% of all disability-adjusted life-years lost in KSA [3]. Ischemic heart disease (IHD), stroke, type 2 diabetes, chronic obstructive pulmonary disease (COPD), chronic kidney disease (CKD), dementia, depression, osteoarthritis, colorectal cancer, and breast cancer accounted for 42.9% of this disability-adjusted life-year burden. In 2011, spending on a similar selection of key NCDs was estimated to account for USD 18.3 billion, or 72.8% of the KSAs total health expenditure [4, 5]. This was equivalent to 2.7% of KSAs gross domestic product in the same year.

These NCDs are also likely to be amenable to public health intervention in KSA. The population in KSA exhibits a high prevalence of NCD risk factors including obesity, tobacco use, physical inactivity, and poor diet. For example, a recent World Bank report on NCDs in KSA indicates between 54.1 and 70.2% of adult females and between 57.5 and 68.3% of adult men are overweight [6]. In 2021, it is estimated that 26.5% of adult men in KSA were using tobacco daily [7]. In the 2013 Saudi Health Interview Survey of 10,735 individuals aged ≥ 15 years, dietary guideline recommendations were met by only 5.2% of individuals for fruits, 7.5% for vegetables, 31.4% for nuts, and 44.7% for fish [8]. In the same survey, 46.5% of women reported being physically inactive [9].

With the growing size of the elderly population, and high prevalence of NCD risk factors, the prevalence of NCDs in KSA will increase leading to a commensurate rise in demand for medical care. We aimed to quantify the growing NCD burden in KSA between 2020 and 2030, and the impact this will have on the national health budget. Our findings will allow KSA to plan for these changes, emphasise the importance of prevention and promotion health policy, and ensure health resources remain adequate into the future.

## Methods

### Priority non-communicable diseases

IHD, stroke, type 2 diabetes, COPD, CKD, dementia, depression, osteoarthritis, colorectal cancer, and breast cancer were chosen as our priority NCDs based on their large contribution to the disability-adjusted life-year burden in KSA, amenability to public health intervention, and the availability of well-defined cost-of-illness estimates.

### Prevalence projections

All priority NCD prevalence projections were based on the population of KSA aged ≥ 15 years. Males and females were split into 5-year age brackets (up to age 85 years with age greater than 85 years considered a single age bracket (i.e., 16–20, 21–25, 26–30, 31–35, 36–40, 41–45, 46–50, 51–55, 56–60, 61–65, 66–70, 71–75, 76–80, 81–85, and > 85 years old). Age- and sex-specific estimates of priority NCD prevalence were taken from the Global Burden of Disease which uses a combination of health data from hospitals, governments, surveys, and other databases around the world and modeling tools to generate estimates for locations and years where data are not available [3]. Prevalence estimates for 2020–2030 were generated using a least squares linear projection of Global Burden of Disease estimates for 2015–2019. Our projected age- and sex-specific prevalence estimates were then multiplied by the age- and sex-specific United Nations population projections [10] for years 2020–2030 to derive the absolute number of people in KSA who may seek care per condition per year.

### Treatment coverage

Treatment coverage (i.e., the proportion of people needing treatment for an illness who receive treatment) rates were applied to the projected prevalence estimates for each priority NCD to calculate the number of patients incurring treatment costs for that condition. These were determined based on the severity of the NCD, the ability to detect cases, and the probability that patients with the condition will seek care. Based on these factors, and consistent with previous estimates, [11–18] the following treatment coverage rates were established: 80% for IHD, stroke, diabetes, COPD, dementia, osteoarthritis, colorectal cancer, and breast cancer; 30% for depressive disorders; and 10% for CKD (see Table 1).

**Table 1:** Treatment coverage and cost-of-illness estimates for priority non-communicable diseases

Non-communicable disease	Treatment coverage	Cost of illness, USD (2020) per person treated per year	Cost of illness reference
Ischemic heart disease	80%	968	[21]
Stroke	80%	2,354	[21]
Diabetes	80%	1,955	[21]
Chronic obstructive pulmonary disease	80%	5,913	[20, 27, 28]
Chronic kidney disease	10%	4,020	[19, 22, 24]
Dementia	80%	33,609	[26]
Depression	30%	4,097	[23]
Osteoarthritis	80%	4,624	[25]
Colorectal cancer	80%	2,682	[21]
Breast cancer	80%	900	[21]

A relatively high treatment coverage target was assigned to IHD, stroke, diabetes, COPD, dementia, osteoarthritis, colorectal cancer, and breast cancer because of the severity of symptoms and ease of detection. On the other hand, people with depression often do not report symptoms or go undiagnosed in primary care settings, [11] and CKD is seriously underdiagnosed in both wealthy and resource-limited countries [12–15]. Therefore, we assigned lower treatment coverage rates for depression and CKD. Treatment coverage rates were assumed to remain constant between 2020 and 2030.

### Cost-of-illness

For each priority NCD, the average cost-of-illness (i.e., the value of the resources that are expended as a result of a health problem) was estimated based on published literature [19–28]. Costs were converted to the equivalent USD amount at the time of the study (if not already in USD) using OANDA Currency Converter [29] and then inflated to 2020 USD using the US Bureau of Labor Statistics Consumer Price Index Inflation Calculator [30]. Our final cost-of-illness estimates are shown in Table 1. Cost-of-illness estimates were assumed to remain constant between 2020 and 2030.

### Budget impact assessment and scenario analyses

Using our projected prevalence, treatment coverage, and cost-of-illness estimates, the budgetary impacts of the ten priority NCDs in KSA were forecast for 2020–2030. The base-case model adopted the assumptions described above. The impact of changes to the base-case model, coming into effect from 2023 onwards, were also explored. These scenarios were designed to depict the consequences of realistic changes in economic or health policy (e.g., drug price changes, healthcare worker salary changes, changes to public/private healthcare ratio, scale up of NCD screening, implementation of tobacco control and healthy eating initiatives). Scenarios evaluated included a 10% reduction in prevalence for priority diseases with a 2023 prevalence > 1% (i.e., IHD, stroke, diabetes, COPD, CKD, depression, and osteoarthritis); a 10% increase in treatment coverage for low treatment coverage priority diseases (i.e., CKD and depression); and a 10% increase or decrease in the costs associated with NCD management. Each scenario was evaluated individually. No combination of scenarios was considered.

## Results

### Projected prevalence

The projected prevalence of priority NCDs in KSA between 2020 and 2030 is shown in Table 2. Over this period, the largest changes in annual prevalence were for CKD, diabetes, and osteoarthritis with increases of 1.9, 1.5, and 1.2 million cases, respectively. The prevalence estimates for colorectal cancer and stroke were estimated to almost double between 2020 and 2030 (97% and 88% increase, respectively). The only priority NCD prevalence projected to increase by less than 60% between 2020 and 2030 was for depression (22% increase).

**Table 2:** Annual prevalence of priority non-communicable diseases among people aged ≥ 15-years, 2020–30

Non-communicable disease	2020	2021	2022	2023	2024	2025	2026	2027	2028	2029	2030
Ischemic heart disease	1,013,588 (3.9%)	1,077,936 (4.0%)	1,142,112 (4.2%)	1,206,115 (4.4%)	1,269,947 (4.5%)	1,333,606 (4.7%)	1,414,888 (4.9%)	1,495,915 (5.1%)	1,576,688 (5.3%)	1,657,207 (5.4%)	1,737,471 (5.6%)
Stroke	529,102 (2.0%)	569,356 (2.1%)	610,422 (2.2%)	652,300 (2.4%)	694,989 (2.5%)	738,491 (2.6%)	787,472 (2.7%)	837,331 (2.8%)	888,068 (3.0%)	939,683 (3.1%)	992,177 (3.2%)
Diabetes	2,690,257 (10.3%)	2,824,930 (10.6%)	2,961,859 (10.9%)	3,101,043 (11.2%)	3,242,481 (11.6%)	3,386,176 (11.9%)	3,545,963 (12.2%)	3,708,341 (12.6%)	3,873,307 (12.9%)	4,040,862 (13.2%)	4,211,007 (13.6%)
Chronic obstructive pulmonary disease	472,947 (1.8%)	498,917 (1.9%)	525,197 (1.9%)	551,786 (2.0%)	578,685 (2.1%)	605,893 (2.1%)	638,371 (2.2%)	671,273 (2.3%)	704,600 (2.3%)	738,352 (2.4%)	772,528 (2.5%)
Chronic kidney disease	3,089,066 (11.8%)	3,245,987 (12.2%)	3,405,431 (12.5%)	3,567,398 (12.9%)	3,731,887 (13.3%)	3,898,898 (13.7%)	4,103,037 (14.1%)	4,310,181 (14.6%)	4,520,328 (15.1%)	4,733,478 (15.5%)	4,949,633 (16.0%)
Dementia	90,854 (0.3%)	96,567 (0.4%)	102,299 (0.4%)	108,052 (0.4%)	113,825 (0.4%)	119,617 (0.4%)	128,207 (0.4%)	136,827 (0.5%)	145,475 (0.5%)	154,153 (0.5%)	162,861 (0.5%)
Depression	1,130,882 (4.3%)	1,154,358 (4.3%)	1,177,957 (4.3%)	1,201,679 (4.4%)	1,225,522 (4.4%)	1,249,488 (4.4%)	1,274,907 (4.4%)	1,300,476 (4.4%)	1,326,195 (4.4%)	1,352,064 (4.4%)	1,378,083 (4.4%)
Osteoarthritis	1,744,566 (6.7%)	1,852,225 (6.9%)	1,960,589 (7.2%)	2,069,659 (7.5%)	2,179,434 (7.8%)	2,289,914 (8.0%)	2,411,937 (8.3%)	2,534,755 (8.6%)	2,658,371 (8.9%)	2,782,782 (9.1%)	2,907,989 (9.4%)
Colorectal Cancer	19,949 (0.1%)	21,592 (0.1%)	23,286 (0.1%)	25,032 (0.1%)	26,830 (0.1%)	28,679 (0.1%)	30,701 (0.1%)	32,775 (0.1%)	34,902 (0.1%)	37,081 (0.1%)	39,313 (0.1%)
Breast cancer	49,487 (0.2%)	53,030 (0.2%)	56,674 (0.2%)	60,421 (0.2%)	64,270 (0.2%)	68,221 (0.2%)	72,410 (0.2%)	76,699 (0.3%)	81,089 (0.3%)	85,580 (0.3%)	90,170 (0.3%)

All values are displayed as absolute prevalence (percentage of population ≥ 15-years-old)

### Budget impact assessment

**Fig. 1:**
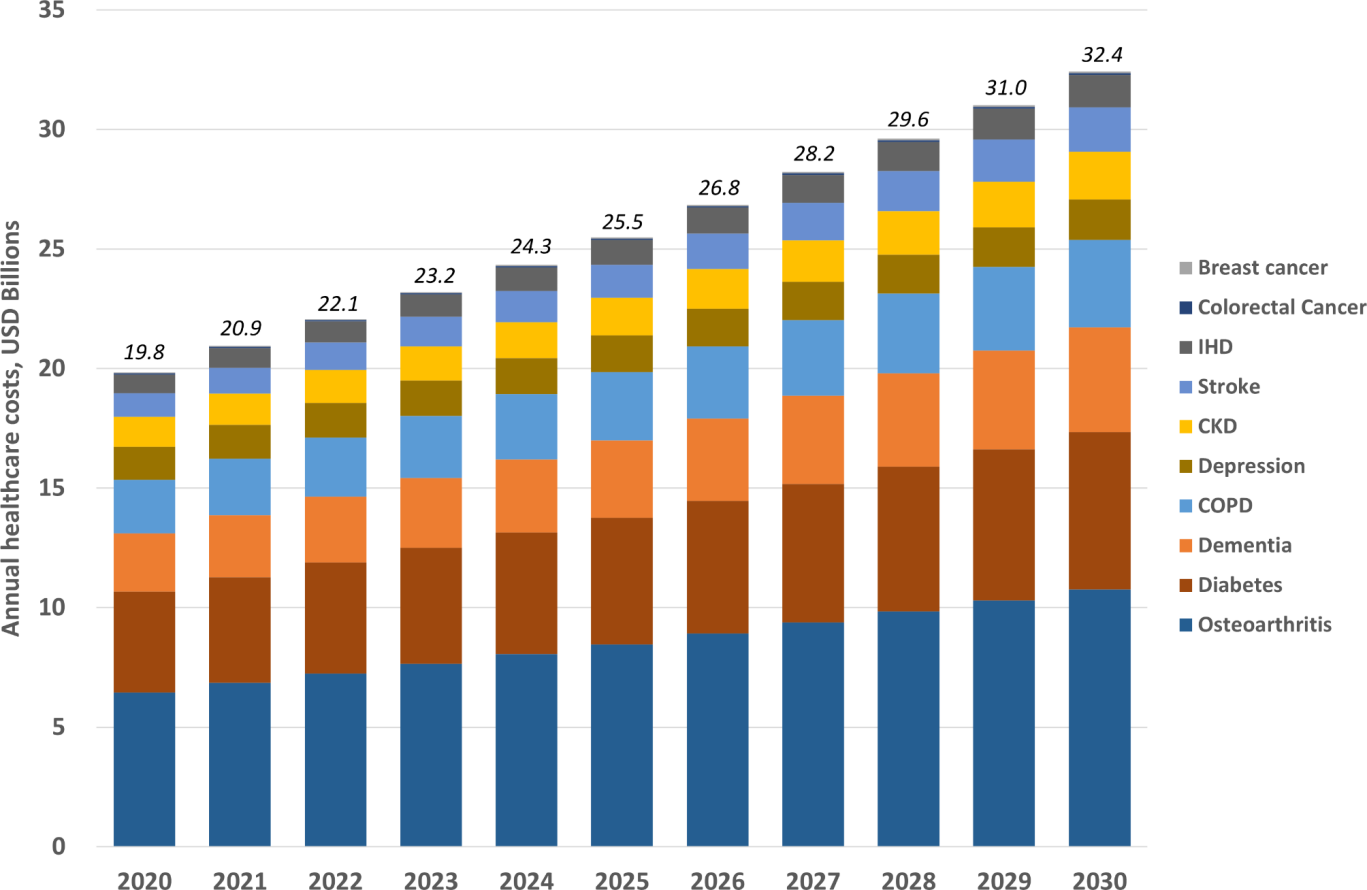
Annual healthcare costs for priority non-communicable diseases among people aged ≥ 15-years, 2020–30. Values at top of columns represent the annual sum for all priority non-communicable diseases. IHD, ischemic heart disease; CKD, chronic kidney disease; COPD, chronic obstructive pulmonary disease

Under our base-case assumptions, it is estimated that the total cost of managing priority NCDs in KSA would increase from USD 19.8 billion in 2020 to USD 32.4 billion in 2030 (an increase of USD 12.6 billion or 63%; Fig. 1 and Supplementary Table 1). The largest USD value increases were projected for osteoarthritis (USD 4.3 billion), diabetes (USD 2.4 billion), and dementia (USD 1.9 billion).

### Scenario analyses

With a 10% change in the cost of illness for all priority NCDs, our 2030 projection was estimated to drop to as low as USD 29.2 billion or increase to as much as USD 35.7 billion. A 10% increase in treatment coverage for CKD caused the base-case 2030 cost projection to increase by USD 2.0 billion (to USD 34.4 billion). An equivalent increase in treatment coverage for depression resulted in an increase of USD 0.6 billion (to USD 33.0 billion). Reductions in disease prevalence for osteoarthritis and diabetes had the largest impact on projected costs for 2030 – a 10% reduction in osteoarthritis prevalence equated to a USD 1.1 billion reduction from base-case (to USD 31.3 billion) and a 10% reduction in diabetes equated to a USD 0.7 billion reduction from base-case (to USD 31.8 billion). Table 3 shows the results for all scenarios evaluated.

**Table 3:** Scenario analyses for total annual healthcare costs for priority non-communicable diseases among people aged ≥ 15-years, 2023-30

Scenario	2023	2024	2025	2026	2027	2028	2029	2030
Base-case	23,192,209,970	24,333,834,297	25,486,341,757	26,848,077,253	28,222,638,570	29,610,025,708	31,010,238,665	32,423,277,442
**Scenarios decreasing costs (in ascending order of 2030 costs)**								
10% reduction in cost of non-communicable disease	20,872,988,973	21,900,450,868	22,937,707,581	24,163,269,528	25,400,374,713	26,649,023,137	27,909,214,798	29,180,949,698
10% reduction in osteoarthritis prevalence	22,426,601,814	23,527,618,213	24,639,256,858	25,955,853,696	27,284,981,829	28,626,641,257	29,980,831,980	31,347,553,998
10% reduction in diabetes prevalence	22,707,206,914	23,826,710,195	24,956,743,897	26,293,488,567	27,642,654,109	29,004,240,524	30,378,247,811	31,764,675,970
10% reduction in chronic obstructive pulmonary disease prevalence	22,931,193,130	24,060,093,340	25,199,730,316	26,546,102,395	27,905,099,466	29,276,721,528	30,660,968,581	32,057,840,626
10% reduction in chronic kidney disease prevalence	23,048,800,581	24,183,812,459	25,329,606,068	26,683,135,156	28,049,369,313	29,428,308,540	30,819,952,837	32,224,302,203
10% reduction in stroke prevalence	23,069,368,905	24,202,953,908	25,347,269,139	26,699,780,578	28,064,952,448	29,442,784,751	30,833,277,485	32,236,430,651
10% reduction in depression prevalence	23,044,511,659	24,183,205,351	25,332,767,129	26,691,378,419	28,062,797,081	29,447,023,115	30,844,056,522	32,253,897,301
10% reduction in ischemic heart disease prevalence	23,098,808,407	24,235,489,637	25,383,067,339	26,738,508,356	28,106,794,896	29,487,926,960	30,881,904,548	32,288,727,660
**Scenarios increasing costs (in descending order of 2030 costs)**								
10% increase in cost of non-communicable disease	25,511,430,966	26,767,217,727	28,034,975,932	29,532,884,979	31,044,902,428	32,571,028,278	34,111,262,531	35,665,605,186
10% increase in chronic kidney disease treatment coverage	24,626,303,858	25,834,052,684	27,053,698,639	28,497,498,229	29,955,331,143	31,427,197,381	32,913,096,944	34,413,029,832
10% increase in depression treatment coverage	23,684,537,671	24,835,930,784	25,998,257,182	27,370,406,703	28,755,443,536	30,153,367,682	31,564,179,140	32,987,877,911

Scenarios were assumed to begin from 2023. All values are in 2020 USD

## Discussion

Managing the growing NCD burden in KSA’s aging population will require substantial increases in healthcare spending over the coming years. We estimated the cost of managing priority NCDs in 2020 was USD 19.8 billion(~ 49% of KSA’s health budget, or 2.7% of KSA’s Gross Domestic Product [4]). This value was projected to increase to USD 32.4 billion by 2030, a 63% increase from costs in 2020. These findings will help KSA plan future health budgets and policy interventions to ensure adequate resources are available to maintain the health of the population.

A global analysis like ours anticipates similarly large increases in NCD costs over time. In 2011, the World Economic Forum estimated that direct and indirect costs would increase globally between 2010 and 2030 by 58% for cancer (from USD 290 billion to USD 458 billion), 20.5% for cardiovascular disease (from USD 863 billion to USD 1.04 trillion), 129% for COPD (from USD 2.1 trillion to USD 4.8 trillion), 49% for diabetes (from USD 500 billion to USD 745 billion), and 140% for mental illness (from USD 2.5 trillion to USD 6.0 trillion) [31]. Further modelling focusing on the negative impacts of NCDs on labor supply and capital accumulation estimates losses in the order of USD 47 trillion worldwide between 2011 and 2030 for cancer, cardiovascular disease, COPD, diabetes, and mental illness [31–33].

We believe a primary-care focused model and multi-sectoral action are needed to promote NCD prevention in KSA. This is envisioned under the New Model of Care program, which is currently being piloted in several areas of KSA [34]. Multi-sectoral action, including working across ministries outside the health sector (e.g., education, media), is also taking shape under the Public Health Authority’s Master Plan for NCD Prevention [6]. Nevertheless, more government funding for preventive and primary care programs is needed. Further monitoring and evaluation are also necessary to ensure the ongoing effectiveness of the abovementioned programs in reducing NCD burden.

There are several limitations to this analysis. Projections, although useful for health policy planning, rely on assumptions. One key assumption is that the parameter data used are accurate. We have used the best pubished data available, along with reasoned assumptions, in order to establish our model parameters. Our models are based on prevalence, treatment coverage, and cost estimates for priority NCDs. This approach may yield different conclusions than one that attempts to enumerate parameter estimates for every NCD. We used a cost-of-illness approach for our budget impact assessment. While this is a commonly used method that sums direct and indirect costs, it is important to acknowledge that the process of estimating cost-of-illness is vulnerable to double counting associated with comorbidities because data on personal medical care costs rarely divide those costs by condition [31]. Therefore, our budget impact results may be overestimated for some or all priority NCDs.

## Conclusion

The number of people aged greater than 65 years per 100 people aged 20–64 years is expected to almost double in KSA between 2020 and 2030. We estimate that KSA will need to substantially increase healthcare spending in the coming years to manage the growing burden of NCDs among its aging population. Planning for future health budgets and policy interventions to maintain the health of the population has to be done now.

### Electronic supplementary material

Below is the link to the electronic supplementary material.

Supplementary Material 1

## Data Availability

The data used for the current study are available from the corresponding author upon reasonable request.

## Abbreviations

KSA: The Kingdom of Saudi Arabia
NCD: Non-communicable disease
IHD: Ischemic heart disease
COPD: Chronic obstructive pulmonary disease
CKD: Chronic kidney disease
