# Supplementary material for: Projected impact of population aging on non-communicable disease burden and costs in the Kingdom of Saudi Arabia, 2020–2030
Source: BMC Health Serv Res. 2023 Dec 8;23:1381. doi: 10.1186/s12913-023-10309-w (PMC10709902; doi:10.1186/s12913-023-10309-w)
Supplement: Supplementary file 1 — Supplementary Material 1 [file 12913_2023_10309_MOESM1_ESM.docx]

**Supplementary Material for: Projected impact of population aging on non-communicable disease burden and costs in the Kingdom of Saudi Arabia, 2020-2030**

**Supplementary Table 1 - Annual healthcare costs for priority non-communicable diseases among people aged ≥15-years, 2020–30**

| **NCD** | **2020** | **2021** | **2022** | **2023** | **2024** | **2025** | **2026** | **2027** | **2028** | **2029** | **2030** |
| --- | --- | --- | --- | --- | --- | --- | --- | --- | --- | --- | --- |
| IHD | 784,922,169 | 834,753,405 | 884,451,223 | 934,015,624 | 983,446,607 | 1,032,744,173 | 1,095,688,976 | 1,158,436,742 | 1,220,987,471 | 1,283,341,164 | 1,345,497,819 |
| Stroke | 996,405,167 | 1,072,211,281 | 1,149,546,439 | 1,228,410,641 | 1,308,803,888 | 1,390,726,179 | 1,482,966,760 | 1,576,861,222 | 1,672,409,567 | 1,769,611,794 | 1,868,467,904 |
| Diabetes | 4,207,561,811 | 4,418,190,954 | 4,632,347,203 | 4,850,030,560 | 5,071,241,023 | 5,295,978,593 | 5,545,886,864 | 5,799,844,612 | 6,057,851,837 | 6,319,908,540 | 6,586,014,720 |
| COPD | 2,237,226,811 | 2,360,077,013 | 2,484,390,874 | 2,610,168,394 | 2,737,409,572 | 2,866,114,410 | 3,019,748,584 | 3,175,391,046 | 3,333,041,795 | 3,492,700,832 | 3,654,368,155 |
| CKD | 1,241,804,384 | 1,304,886,887 | 1,368,983,388 | 1,434,093,888 | 1,500,218,386 | 1,567,356,883 | 1,649,420,975 | 1,732,692,572 | 1,817,171,674 | 1,902,858,280 | 1,989,752,390 |
| Dementia | 2,442,819,700 | 2,596,411,284 | 2,750,542,436 | 2,905,213,154 | 3,060,423,441 | 3,216,173,294 | 3,447,134,051 | 3,678,883,836 | 3,911,422,647 | 4,144,750,485 | 4,378,867,351 |
| Depression | 1,389,966,799 | 1,418,821,773 | 1,447,827,208 | 1,476,983,104 | 1,506,289,460 | 1,535,746,277 | 1,566,988,348 | 1,598,414,897 | 1,630,025,923 | 1,661,821,426 | 1,693,801,407 |
| Osteoarthritis | 6,453,496,830 | 6,851,749,550 | 7,252,611,126 | 7,656,081,556 | 8,062,160,842 | 8,470,848,983 | 8,922,235,576 | 9,376,567,418 | 9,833,844,509 | 10,294,066,847 | 10,757,234,435 |
| Colorectal Cancer | 42,801,596 | 46,327,273 | 49,963,314 | 53,709,718 | 57,566,487 | 61,533,620 | 65,871,853 | 70,322,669 | 74,886,068 | 79,562,050 | 84,350,614 |
| Breast cancer | 35,630,508 | 38,181,289 | 40,805,563 | 43,503,330 | 46,274,590 | 49,119,343 | 52,135,265 | 55,223,557 | 58,384,217 | 61,617,247 | 64,922,647 |
| **SUM TOTAL** | **19,832,635,775** | **20,941,610,708** | **22,061,468,773** | **23,192,209,970** | **24,333,834,297** | **25,486,341,757** | **26,848,077,253** | **28,222,638,570** | **29,610,025,708** | **31,010,238,665** | **32,423,277,442** |

All values are in 2020 USD. NCD, non-communicable disease; IHD, ischemic heart disease; COPD, chronic obstructive pulmonary disease; CKD, chronic kidney disease
